# Vitamin D in Infectious Diseases: A Narrative Review Focusing on COVID-19, Long COVID, and Influenza

**DOI:** 10.3390/nu18040634

**Published:** 2026-02-14

**Authors:** Olga Adriana Caliman-Sturdza, Roxana Elena Gheorghita, Iuliana Soldanescu, Mihai Dimian, Serghei Mangul

**Affiliations:** 1 Faculty of Medicine and Biological Sciences, Stefan cel Mare University of Suceava, 720229 Suceava, Romania; olga.caliman-sturdza@usm.ro (O.A.C.-S.);; 2 Suceava Emergency Clinical County Hospital, 720224 Suceava, Romania; 3 Integrated Center for Research, Development, and Innovation for Advanced Materials, Nanotechnologies, Manufacturing and Control Distributed Systems (MANSiD), Stefan cel Mare University of Suceava, 720229 Suceava, Romania; iuliana.soldanescu@usm.ro (I.S.); dimian@usm.ro (M.D.); 4 Department of Computers, Electronics and Automation, Stefan cel Mare University of Suceava, 720229 Suceava, Romania; 5 Department of Clinical Pharmacy, USC Alfred E. Mann School of Pharmacy and Pharmaceutical Sciences, University of Southern California (USC), Los Angeles, CA 90033, USA; 6 Department of Quantitative and Computational Biology, USC Dornsife College of Letters, Arts and Sciences, University of Southern California (USC), Los Angeles, CA 90089, USA

**Keywords:** vitamin D, immunological mechanisms, SARS-CoV-2, post-acute sequelae of COVID-19, influenza

## Abstract

Vitamin D is a secosteroid hormone traditionally recognized for its role in bone and mineral metabolism, but it is increasingly understood to also function as an important immunomodulator influencing susceptibility to and outcomes of infectious diseases. This narrative review summarizes current evidence on the immunological, clinical, and preventive effects of vitamin D in the context of novel coronavirus disease (COVID-19), post-acute sequelae of SARS-CoV-2 infection (long COVID), and influenza. Mechanistically, vitamin D enhances innate immune defenses through the induction of antimicrobial peptides, including cathelicidin and defensins, and modulates adaptive immunity by suppressing maladaptive Th1/Th17 responses while promoting regulatory T-cell activity. Observational studies have frequently associated vitamin D deficiency with more severe COVID-19 outcomes; however, these associations may be influenced by confounding factors and reverse causality. Some meta-analyses suggest that vitamin D supplementation reduced rates of intensive care unit admission and ventilatory support, particularly among older adults and individuals with low baseline serum 25-hydroxyvitamin D concentrations. Emerging evidence also indicates that inadequate vitamin D status may be associated with an increased risk and symptom burden of long COVID, although causality has not been established. In the case of influenza, a limited number of randomized controlled trials (RCTs) and meta-analyses report a modest but statistically significant reduction in infection risk, especially with daily or weekly vitamin D supplementation in populations with low baseline vitamin D levels. Clinical guidelines consistently recommend maintaining adequate vitamin D status for general health but do not endorse high-dose vitamin D as a treatment for COVID-19 due to inconsistent trial findings. Overall, vitamin D should not be considered a standalone therapeutic agent; rather, maintaining sufficient vitamin D levels represents a low-risk, potentially beneficial strategy to support immune resilience against respiratory viral infections.

## 1. Introduction

Vitamin D has traditionally been known for its role in bone and mineral metabolism, but accumulating evidence demonstrates that it also plays an important role in immune regulation and host defense against infections [1,2]. During the novel coronavirus disease (COVID-19) pandemic, interest in vitamin D intensified as researchers explored its potential role in modulating immune responses to severe acute respiratory syndrome coronavirus 2 (SARS-CoV-2) and other respiratory viruses [3]. In addition, attention has increasingly focused on “long COVID”, also referred to as post-acute sequelae of SARS-CoV-2 infection (PASC), which is characterized by persistent or new symptoms lasting weeks to months after the acute phase of infection [4]. Vitamin D deficiency in the population has raised the question of whether maximizing vitamin D levels would be beneficial in viral infections [5]. This narrative review summarizes current understanding of the immunological mechanisms of vitamin D, and it compiles the results of studies on COVID-19 (including long COVID sequelae) and influenza. We bring to the fore important mechanisms, observational and interventional study findings, emerging evidence about long COVID, preliminary influenza prevention evidence, pertinent meta-analyses, guidelines, and controversies.

## 2. Methods

This narrative review was conducted with a focus on the literature published between 2020 and 2025, while incorporating earlier foundational studies to provide relevant historical context. The review included mechanistic studies, observational studies, randomized controlled trials (RCTs), systematic reviews, meta-analyses, and clinical guidelines addressing the role of vitamin D in COVID-19, long COVID, and influenza.

Literature searches were performed using the PubMed database and targeted Google Scholar searches. Search terms included combinations of “vitamin D,” “25-hydroxyvitamin D,” “COVID-19,” “long COVID,” “post-acute sequelae of SARS-CoV-2 infection,” “influenza,” “interventional trials,” and “meta-analyses.” Reference lists of key articles and reviews were also screened to identify additional relevant publications. Search results were screened for relevance to the study question through a two-stage process. First, titles and abstracts were reviewed to exclude clearly irrelevant records. Second, potentially eligible articles underwent full-text assessment.

Studies were included if they were peer-reviewed, published in English, and addressed immunological mechanisms, clinical outcomes, or the preventive effects of vitamin D in relation to COVID-19, long COVID, or influenza. Excluded materials included conference abstracts, commentaries, opinion pieces, general media articles and newsletters.

As this is a narrative review, a formal systematic quality assessment or risk-of-bias scoring was not performed. However, preference was given to higher levels of evidence, including RCTs, meta-analyses, and large cohort studies, when available. Conflicting findings were intentionally included and discussed to reflect the heterogeneity of the existing evidence. The narrative approach allowed for the integration of mechanistic, clinical, and epidemiological data but may be subject to selection bias and does not provide quantitative effect estimates. These limitations should be considered when interpreting the conclusions of this review.

For consistency, vitamin D dosing regimens are described using standardized terminology throughout the manuscript. “Low-dose” supplementation refers to daily doses ≤ 800 IU/day, “moderate-dose” to daily doses of 1000–4000 IU/day, and “high-dose” supplementation to daily doses > 4000 IU/day or equivalent weekly dosing. The term “bolus dosing” refers to the administration of large intermittent doses (≥50,000 IU given as a single or infrequent dose), irrespective of the vitamin D formulation. Serum 25-hydroxyvitamin D concentrations are reported in nmol/L throughout the manuscript (1 ng/mL = 2.5 nmol/L).

## 3. Immunological Mechanisms of Vitamin D in Infections

**Figure 1:**
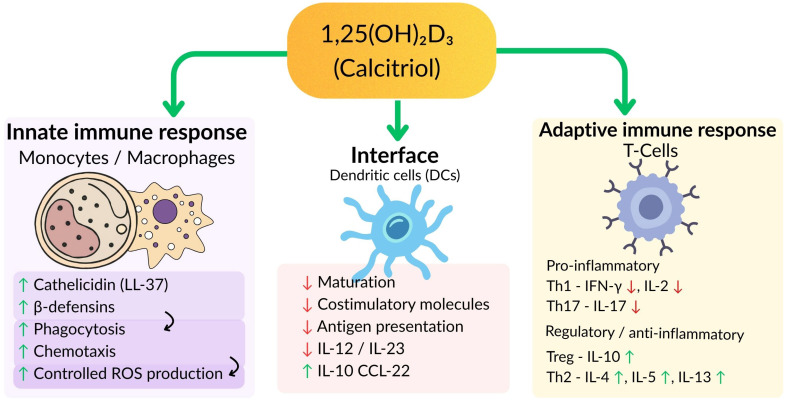
Modulatory effects of the active form of vitamin D, 1,25(OH)_2_D_33_ (calcitriol), on innate and adaptive immune responses. Arrows indicate the direction of modulation: upward arrows (↑) denote upregulation/increase, whereas downward arrows (↓) denote downregulation/decrease. Calcitriol exerts a broad immunomodulatory role, acting simultaneously on innate immunity, on dendritic cells. In monocytes and macrophages, 1,25(OH)_2_D_3_ stimulates the production of the antimicrobial peptides cathelicidin (LL-37) and β-defensins, enhances phagocytic activity and chemotactism, and helps maintain a controlled level of reactive oxygen species, favoring an efficient but well-regulated antimicrobial response. In dendritic cells, calcitriol limits maturation processes and reduces the expression of costimulatory molecules and antigen-presenting capacity, leading to decreased secretion of the proinflammatory cytokines IL-12 and IL-23. Concomitantly, it promotes the secretion of IL-10 and CCL-22, orienting the DC profile towards a tolerogenic one. In adaptive immunity, calcitriol attenuates the differentiation of proinflammatory Th1 (by reducing IFN-γ and IL-2) and Th17 (by decreasing IL-17) subsets, while promoting the development of anti-inflammatory and regulatory responses by increasing Treg activity (IL-10) and promoting Th2 polarization (IL-4, IL-5, IL-13). Through these integrated mechanisms, vitamin D contributes to the maintenance of an appropriate immunological balance, preventing excessive inflammation and supporting defense mechanisms against infection.

Vitamin D is an immunomodulatory hormone that can have an effect on both the innate and adaptive immune system [6] (Figure 1). It attaches to the vitamin D receptor (VDR) on numerous immune cells (e.g., macrophages, dendritic cells, T and B lymphocytes) and changes gene expression and cytokine secretion [7].

Vitamin D enhances the expression of cathelicidin (LL-37) and defensins genes, as well as antimicrobial peptides, which have direct antiviral and antibacterial effects [8]. Such peptides may target the viral replication process and improve the chemotaxis of immune cells (macrophages) to infection sites, which improves the innate immune response at mucosal surfaces [9]. It applies to respiratory viruses because an increase in the level of cathelicidin can enhance mucosal immunity in the lungs and airways [10]. Vitamin D is more likely to alter the adaptive immune response towards a pro-inflammatory phenotype. It can decrease the hyperactive Th1 and Th17 cell responses (producers of inflammatory cytokines such as interleukin (IL)-6, IL-17 and tumor necrosis factor (TNF)-α) and increase the development of Th2 and regulatory T-cell (Tregs) [11]. Such changes can moderate cytokine surges and hyperinflammation in severe infections [12]. For example, in COVID-19 cases, adequate vitamin D may dampen the overproduction of inflammatory molecules that underlies acute respiratory distress syndrome [13]. It has been observed that vitamin D inhibits pro-inflammatory cytokines and enhances the production of anti-inflammatory cytokines and Tregs, which may help prevent immunologically mediated tissue damage [14]. Proper levels of vitamin D have been associated with enhanced activity of innate immune cells. Vitamin D is able to stimulate the microbicidal effect of macrophages and natural killer cells, through both the above antimicrobial peptides and other mechanisms (such as the induction of autophagy in infected cells) [15,16,17,18]. Vitamin D can also reduce the viral multiplication of infected cells through VDR signaling, which remains to be explained [19]. Some research even indicates that vitamin D (and similar molecules, such as lumisterol) may have direct non-genomic antiviral action against some viruses [20]. In addition to activating classical immune cells, vitamin D helps maintain the integrity of epithelial barriers (including in the respiratory tract)—an initial response to inhaled pathogens. It also works with the renin–angiotensin system (RAS): vitamin D suppresses renin and can affect the expression of ACE2 [21]. As SARS-CoV-2 binds to the ACE2 receptor, it has been postulated that the presence of vitamin D may indirectly impact viral entry or alleviate the effect of a virus on RAS dysregulation, although this has yet to be proven [22]. In addition, the role of vitamin D in mitigating risk factors such as hypertension and diabetes (via RAS and insulin sensitivity pathways) has a potential indirect impact on infection outcomes, such as comorbid conditions associated with adverse outcomes of COVID-19 [23,24].

To conclude, vitamin D is a versatile regulator of the immune system, as it can increase the native defenses and suppress the overreactive response to inflammation. This has become the biological basis for studying vitamin D in respiratory infections like COVID-19 and influenza. The in vivo effect of these mechanisms on real infection outcomes relies on intricate factors and has been the topic of active studies in recent years.

## 4. Vitamin D and Influenza

The possible effects of vitamin D on influenza were discovered more than ten years ago, before COVID-19. The seasonal respiratory virus influenza offered some of the first indications of the role of vitamin D in immunity: since influenza outbreaks occurred during the winter, when sunlight (and, therefore, the formation of endogenous vitamin D) is minimal, researchers theorized that the lack of vitamin D may have contributed to the higher rates of susceptibility in winter. The effect of vitamin D on influenza infection or severity has been studied for many years. The immunologic effects of vitamin D mentioned above (the induction of antimicrobial peptides and the regulation of cytokines, as well as the enhancement of innate immunity) are highly relevant to influenza [25]. As a case in point, vitamin D induces cathelicidin LL-37, which may disrupt influenza viruses and infected cells, whereas the anti-inflammatory action of vitamin D may inhibit the extreme lung inflammation observed in influenza pneumonia [26,27]. Animal experiments have indicated that vitamin D metabolites have the potential to decrease lung damage in mice with influenza, and an in vitro experiment indicated that vitamin D could improve epithelial immunity to influenza [28]. The mechanisms in question do not disprove the hypothesis that an adequate amount of vitamin D might not only decrease the risk of developing influenza but also alleviate the symptoms of the disease once contracted [29].

A number of observational studies have attributed incidences of respiratory infections such as influenza to low vitamin D. Individuals with lower vitamin D levels have been observed to contract more respiratory tract infections during the winter season, and in certain studies, higher rates of lab-confirmed influenza were reported in people with lower 25-hydroxyvitamin D (25(OH)D) levels [30]. On the other hand, there were cases of populations with higher vitamin D (through sun exposure or supplements) exhibiting lower influenza rates, but confounding factors (e.g., general health, exposure levels) may also contribute [31]. There are even historical anecdotes that cod liver oil (high in vitamin D) was used as a remedy in outbreaks of influenza in the early 20th century, though controlled data were lacking at the time [32].

The clearest data relates to RCTs of vitamin D supplementation as a preventive against influenza. In a groundbreaking study of Japanese schoolchildren (Urashima et al., 2010), children who received vitamin D_3_ (1200 IU daily) in winter experienced a major reduction in influenza A infection (average reduction of 42 percent) versus placebo [33]. This was a tiny trial that nevertheless directed attention to the potential protective effect of vitamin D. Since then, various RCTs across the globe have been conducted to test vitamin D in several groups of people (children, adults, the elderly) to prevent influenza or influenza-like illness during the flu season [34,35,36]. These individual trials reported both beneficial and neutral effects, possibly because of differences in baseline vitamin D levels, dosing, and the eventual outcome, which was mostly influenza or any respiratory infection.

In order to obtain a better picture, researchers have combined the results of multiple trials. A meta-analysis in *Frontiers in Nutrition* (2022) investigated the effect of vitamin D supplementation on influenza infection in 10 RCTs (N = 4859), which were specifically analyzed [37]. The combined effect indicated that vitamin D supplements had a statistically significant preventive effect: the risk of influenza infection was lower in individuals receiving vitamin D supplements (approximately 22% risk reduction: risk ratio of about 0.78, with the confidence reflecting a 95-percent interval of 0.64 to 0.95) compared with those who did not receive them [37]. It is worth noting that the heterogeneity was low in this analysis, which implied a rather similar effect across studies. The authors concluded that vitamin D plays a small, yet significant role in preventive action against influenza and recommended that public health policies also include the use of vitamin D supplements to prevent influenza infections [37]. This is consistent with an earlier meta-analysis of individual patient data (2017) that concluded that vitamin D specifically conferred protection to those who initially had very low vitamin D levels. Additionally, a daily or weekly regimen was better at preventing acute respiratory infection, including influenza, than infrequent doses [38]. Furthermore, a new 2021 meta-analysis of 46 trials (primarily pre-COVID-19 research on different acute respiratory infections) proved that vitamin D supplementation remains safe and generally significantly lowers the risk of developing respiratory infection (by an average of about 8 percent) [39]. This was more effective in some of the subgroups—trials that used daily doses of 400–1000 IU over a maximum of 12 months, and trials that involved children aged 1–16 years, had higher protection (OR 0.70–0.82) and higher significance of the benefit (OR 0.71) [39]. Since influenza is among the most common respiratory virus types, these results indicate that proper vitamin D intake is a reasonable preventive strategy.

Although one of the interests is infection prevention, other studies have examined whether vitamin D can affect the severity of the influenza illness (e.g., symptoms, complications) should one become infected [40,41]. There is little hard evidence for this, as trials tend to be incidence-based. Nonetheless, it has been suggested that people with adequate vitamin D levels may have less severe disease. For example, a single observational study identified that adults without vitamin D and with influenza were more likely to develop complications such as pneumonia [42]. In addition, the immune-modulating effect of vitamin D may help avoid the overreactive response to inflammation, which is a possible cause of serious influenza (similar to the cytokine storms with COVID-19) [43,44]. In a small trial in adults with new influenza infection, high-dose vitamin D testing as an adjunct therapy demonstrated trends of faster fever and inflammation resolution, but did not show statistical significance due to the size of the sample [45,46,47].

In general, the advantages of vitamin D in influenza, mostly in the prevention of susceptibility, are supported by the evidence. Vitamin D has the potential to become an immune support helping to address deficiencies in innate immunity and suppressing uncontrolled inflammation in the host when exposed to influenza, and it shifts the odds in favor of the host. Public health experts have taken note: vitamin D supplementation, especially in winter, is often recommended as a general measure to support immune health (which would include protection against seasonal flu) [48]. It should be noted that vitamin D should not substitute interventions such as influenza vaccination; instead, it is one support method among many. The reliability of the protective signal in meta-analyses is promising and indicates that adequate vitamin D levels in the population (achieved by diet, supplements or sunlight) could modestly reduce the annual flu burden [19,29,49]. This especially applies to high-risk populations, including the elderly and chronically ill, who not only tend to be deficient in vitamin D but are also at risk of severe influenza [49].

## 5. Vitamin D RCTs for Influenza Prevention/Treatment

Several RCTs were conducted to examine vitamin D supplementation and its preventive or therapeutic effects on influenza. These consist of published peer-reviewed trials in a variety of populations (infants, children, adults, elderly, healthcare workers, and special patient groups) and registered clinical trials (some of which are completed; others are ongoing). A summary table of key RCTs, with trial identifiers (where available), settings, design, and outcomes, is shown below (Table S1).

The results of each trial have to be taken in context, as some of them (e.g., those among children and infants) indicated a protective effect of vitamin D against influenza [33,50], while others suggested no benefit [51,52] or even a potential damaging effect with intermittent high doses [46]. It is also important to note that vitamin D seems to have a more positive effect on non-influenza respiratory diseases in a number of studies [51,53]. Current studies are also attempting to understand the role of vitamin D in preventing influenza, and the findings of recent clinical trials (such as NCT04810949) are expected in the future [54].

Although some randomized trials and meta-analyses report a modest reduction in influenza risk with vitamin D supplementation, several well-conducted trials have shown no significant effect, particularly in populations replete in vitamin D or when intermittent high-dose regimens were used [55]. Overall, the influenza literature supports, at most, a small preventive effect, with substantial variability across studies.

## 6. Vitamin D and COVID-19 Outcomes

While the evidence linking vitamin D to influenza outcomes is largely derived from pre-pandemic studies and relatively homogeneous trial designs, the emergence of SARS-CoV-2 prompted a rapid expansion of observational and interventional research in a far more complex and heterogeneous clinical setting. Many observational studies were conducted on the relationship between the COVID-19 outcomes of patients and their vitamin D levels during the pandemic. In general, most (but not all) studies reported that low levels of 25(OH)D were associated with increased susceptibility to SARS-CoV-2 infection or adverse COVID-19 outcomes [56,57,58,59,60,61,62]. An illustrative example is a comparison study of COVID-19 patients admitted to hospitals and healthy controls in China, which demonstrated much lower median levels of vitamin D in COVID-19 patients and showed that vitamin D deficiency (e.g., 25(OH)D < 30 nmoI/L) is associated with more severe disease [60]. Severe COVID-19 is defined in accordance with commonly used clinical trial and guideline criteria, including the need for hospitalization with supplemental oxygen, admission to an intensive care unit, requirement for mechanical ventilation, or COVID-19-related mortality [63].

Similarly, several retrospective cohorts have indicated that COVID-19 patients with vitamin D deficiency had greater rates of hospital admission, intensive care unit (ICU) admission, and mortality compared to those with adequate vitamin D levels [62,64,65]. The result of a systematic review (17 observational studies, total N = approximately 2756) led to the conclusion that patients who were vitamin D-deficient had a higher risk of mortality, extended hospitalization time, and more severe COVID-19 than patients who were not deficient [66]. Moreover, population studies have reported that people who consistently used vitamin D supplements before the pandemic had a low chance of testing positive for COVID-19 or developing serious consequences [67,68]. These patterns suggest a potential protective association between higher vitamin D levels and a better COVID-19 prognosis. However, observational findings have not been unanimous. Many of them did not observe an independent relationship between baseline vitamin D levels and risk of COVID-19 infection after controlling for confounding factors [69,70]. An excellent example is the analysis of the United Kingdom Biobank (n = 348,000), which showed no significant difference in post-pandemic 25(OH)D and later SARS-CoV-2 infection when accounting for other variables, such as age, obesity, and ethnicity [71]. Herein lies the issue of confounding: lower vitamin D levels are associated with population groups (e.g., older, obese, or having comorbid illnesses) who are more at risk of COVID-19; hence, it may be difficult to separate cause and effect [72]. In addition, the severity of the disease itself could affect vitamin D status; extreme inflammation can reduce the level of 25(OH)D in the circulation (perhaps through tissue breakdown or acute phase response) [73]. An experimental study indicated that an acute inflammatory stimulus may reduce vitamin D levels several hours later, which suggest that COVID-19 might lead to low vitamin D levels and not vice versa [73,74]. Because acute infection and systemic inflammation may influence circulating 25-hydroxyvitamin D concentrations, the possibility of reverse causality cannot be excluded, particularly in studies measuring vitamin D levels during or after acute illness [73].

Still, regardless of these subtleties, the general observation data are inclined towards a correlation between poor vitamin D levels and adverse outcomes of COVID-19 [75,76]. For example, a meta-analysis of 21 observational studies identified a statistically significant difference in the mean levels of vitamin D between COVID-19 patients (and particularly severe cases) and uninfected controls, although that analysis did not find a statistically significant difference in existing infections or deaths when combining all studies [77]. Moreover, some ecological studies noted higher COVID-19 incidence or mortality in populations at higher latitude or with known widespread vitamin D deficiency, though such studies are hypothesis-generating at best [78]. Overall, a lack of vitamin D in COVID-19 patients, especially those with severe disease, has been identified, and previous deficiency in vitamin D seems to be an independent variable in the context of more adverse results in numerous studies [73,79,80,81]. However, these data cannot be used to conclude causality because of confounding and reverse causation. They provide a powerful push towards clinical trials aimed at establishing whether vitamin D supplementation would positively affect COVID-19 outcomes.

To shift correlation to causation, scientists initiated RCTs and intervention studies with the aim of studying the effects of vitamin D supplementation on patients with or at risk of COVID-19. The trials have been mixed in design: some of them administered high doses of vitamin D to patients in the hospital to determine whether the severity of the disease is reduced; others administered vitamin D in the form of daily doses to determine whether the rate of infection could be reduced. Individual trial results have been inconclusive and a number of systematic reviews have compiled the evidence. Early small trials suggested a benefit in specific settings. A pilot RCT in Córdoba, Spain (2020), has found that high-dose calcifediol (25(OH)D), when used in hospitalized COVID-19 patients, significantly lowered the number of ICU admissions compared with controls, which led to the conclusion of a potential acute effect of deficiency correction [82]. However, in later, larger studies, less encouraging or null findings were found. For example, a strict RCT in Brazil (Murai et al., JAMA 2021) provided one high dose (200,000 IU) of vitamin D_3_ to hospitalized moderate-to-severe COVID-19 patients and reported no significant decrease in either both length of stay or other primary outcomes compared to placebo [83]. The results of other hospital-based trials have been heterogeneous regardless of dosing regimen and sample size, and some of these reported an improvement in inflammatory markers or oxygen requirements, with others not demonstrating a clear clinical benefit [71,84,85].

Some trials were performed to determine whether vitamin D would prevent COVID-19 or reduce outpatient illness. A Mexican RCT among frontline healthcare workers found that 4000 IU/day of vitamin D_3_ for 30 days markedly reduced PCR-confirmed infection compared with placebo in a small cohort [86], whereas a large Norwegian trial (>34,000 participants) using cod liver oil providing ~400 IU/day vitamin D (plus other nutrients) reported no difference in infection or disease severity; the low event rate and modest dosing may have limited the ability to detect an effect [87]. Together, these findings suggest that the prophylactic benefit—if present—may depend on baseline deficiency prevalence, exposure risk, dose, and duration. Meta-analyses integrating RCTs and observational evidence reflect this overall pattern: observational studies often suggest stronger associations than randomized evidence. A systematic review compiling eight RCTs and eight cohort studies (total N = 3359 COVID-19 patients) reported that pooled RCT data did not show a significant reduction in mortality (RR = 0.94, 95% CI = 0.69–1.29) or a significant effect on ICU admission or mechanical ventilation, whereas cohort data suggested a much larger association with reduced mortality among supplemented patients (RR = 0.33, 95% CI = 0.23–0.47), highlighting frequent discrepancies between study designs and potential residual confounding in non-randomized data [65]. A more comprehensive and recent synthesis (Sartini et al., *Nutrients*, 2024) incorporating 21 RCTs and additional observational studies reported more favorable signals for some outcomes: vitamin D supplementation was associated with reduced ICU admission in the overall analysis (pooled OR ≈ 0.55), and RCT-only analyses suggested reduced odds of intubation/mechanical ventilation (reported as OR = −0.50, 95% CI = −0.27 to −0.92) [88]. Effects on mortality were less consistent, with observational studies showing stronger reductions than RCTs [88]. Subgroup analyses suggested a greater benefit in older patients and those with severe disease—groups more likely to be deficient and at higher baseline risk—though heterogeneity across trials remained substantial [51]. In summary, intervention evidence remains mixed. Some trials and meta-analyses support clinically meaningful improvements (particularly reduced ICU admission or ventilation) in selected populations [89,90], while others show minimal or no effect on primary endpoints such as mortality or length of hospital stay [91]. A plausible reason for this is that baseline vitamin D status is a key effect-modifier: individuals with marked deficiency may benefit most from supplementation, while high-dose vitamin D may confer limited added value to patients with sufficient levels [3]. Importantly, vitamin D is generally safe and may be a reasonable adjunct to correct deficiency, but it should not be framed as a substitute for established COVID-19 prevention or treatment strategies [92]. This balanced interpretation aligns with clinical guidance, as discussed later. Randomized trials of vitamin D or calcifediol for COVID-19 treatment and prevention (published and key registered/ongoing studies) are summarized in Table S2.

Importantly, the heterogeneity of findings across intervention studies is likely driven by substantial methodological differences, including the vitamin D formulation, dosing strategy, timing of supplementation, and baseline vitamin D status of the participants. Trials using calcifediol, which rapidly increases circulating 25(OH)D concentrations, have more consistently reported favorable clinical signals compared with trials using cholecalciferol, particularly when supplementation was initiated early in the disease course [93]. In contrast, several large trials administered high-dose cholecalciferol after hospitalization, often without stratification by baseline 25(OH)D levels, potentially limiting the capacity to detect a benefit [94]. Accumulating evidence suggests that individuals with low baseline 25(OH)D concentrations derive the greatest potential benefit, whereas supplementation in populations replete in vitamin D may yield minimal clinical effects. These factors likely substantially contribute to the inconsistency observed across randomized trials and meta-analyses.

A critical limitation of many intervention studies is the lack of participant stratification according to baseline and achieved serum 25(OH)D concentrations. Several trials enrolled participants with heterogeneous or largely sufficient baseline vitamin D levels and did not analyze outcomes according to achieved post-supplementation 25(OH)D status [95]. This methodological approach may obscure potential benefits in deficient individuals, as supplementation is unlikely to yield measurable clinical effects in participants who are already replete in vitamin D. Moreover, failure to confirm adequate correction of deficiency following supplementation limits the interpretation of null results. Taken together, insufficient stratification by baseline and attained 25(OH)D concentrations likely substantially contributes to the inconsistent findings reported across randomized controlled trials and meta-analyses [96]. While several early or small-scale trials suggested potential benefits of vitamin D supplementation in COVID-19, the majority of larger, well-controlled randomized trials have reported null findings for primary clinical outcomes, including mortality and length of hospital stay [97]. Notably, trials administering high-dose cholecalciferol to hospitalized patients after disease onset have generally failed to demonstrate significant benefit. These null results weigh heavily in the overall interpretation of the evidence and indicate that vitamin D supplementation does not provide consistent therapeutic effects in unselected COVID-19 populations [98]. Positive signals observed in selected trials and subgroup analyses should therefore be interpreted cautiously and considered exploratory rather than definitive.

## 7. Emerging Research: Vitamin D and Long COVID

The pandemic’s resolution shifted the focus to long COVID (or post-acute sequelae of COVID-19, PASC), the syndrome in which the symptoms endure for weeks or months after acute infection. Researchers are starting to examine the possibility of vitamin D status affecting the onset or intensity of long COVID due to the immunological and anti-inflammatory effects of vitamin D. Emerging observational evidence suggests an association between low vitamin D status and an increased risk or symptom burden of long COVID; however, causal relationships have not been established. Notably, a 2023 study (di Filippo et al., J. Clin. Endocrinol. Metab.) evaluated 100 COVID-19 survivors 6 months after hospitalization, half of whom met the criteria for long COVID and half of whom did not (matched controls) [63]. The long COVID patients had significantly lower serum 25(OH)D levels on follow-up (median 20.1 ng/mL) compared to those without long-term symptoms (23.2 ng/mL) [99]. Furthermore, among patients who were vitamin D-deficient at the time of their illness and remained deficient, those with long COVID had especially low follow-up levels (average 12.7 ng/mL) versus those without long COVID (15.2 ng/mL) [99]. In multivariate analysis, a lower vitamin D level at follow-up was the only significant predictor of long COVID in that cohort (each 1 ng/mL decrease in 25(OH)D was associated with a 9% increase in the odds of long COVID). The authors concluded that vitamin D deficiency might be a risk factor for long COVID and recommended that vitamin D levels of COVID-19 patients be checked and restored after discharge.

A larger prospective study of Thai patients with mild COVID-19 and 3+ months follow-up reported results more recently (Matangkha et al., 2025). Among this group, approximately 65% experienced some form of long COVID syndrome (in accordance with the loose definition of the latter as any type of ongoing symptom) [100]. The median 25(OH)D level of patients who eventually developed long COVID was 21.5 ng/mL as opposed to 25.5 ng/mL in patients who did not [101]. A strong independent risk factor was also vitamin D deficiency (defined here as <20 ng/mL): patients with sufficient levels had odds of long COVID that were about 5.8 times lower than those who were deficient (adjusted OR 5.80, 95% CI 2.10–16.13) [100]. Moreover, vitamin D deficiency was also linked to = more enduring symptoms in various organ systems (the adjusted incidence rate ratio of the number of long COVID symptoms was reported to be about 3.3). Generalized, respiratory (e.g., persistent cough or breathlessness) and dermatologic problems were the most frequent long COVID symptoms in this mild cohort [101]. Using these results, the authors proposed that adequate vitamin D (with the help of supplement intakes and safe exposure to sunlight) in COVID patients could lower this risk and the severity of long-term sequelae.

These investigations, in conjunction with other reports, all point to the trend of individuals with lower levels of vitamin D being more likely to experience long COVID or more severe long-term symptoms [100,102,103]. Scientists assume that the established immunoregulatory properties of vitamin D may contribute to recovery by overcoming inflammation and promoting tissue repair following acute infection [104,105,106]. On the other hand, deficiency may predispose one to abnormal immune recovery, which could be involved in long-term symptoms (as lingering events of inflammatory or autoimmune response) [74]. This is partially mechanistically supported by the fact that it has been observed that vitamin D deficiency is connected to an increase in inflammatory markers and increased immune resolution, which might easily result in the pathology of long COVID [107,108].

It should be mentioned that the existing evidence is largely observational; therefore, we cannot draw conclusions about causation. Intervention trials are necessary to produce evidence on whether the use of vitamin D supplementation post-COVID-19 would help prevent the effects of long COVID or accelerate recovery. These trials are now under consideration. For example, a prospective trial will test combined vitamin D and magnesium therapy in patients with long COVID to determine whether it enhances symptoms (magnesium is a cofactor to vitamin D activation) [109,110]. Researchers generally agree that laboratory testing of vitamin D levels in patients with persistent post-COVID symptoms is rational, and preventing deficiency is a wise step at this point due to the overall health benefits of vitamin D [111].

So far, a number of RCTs have been conducted to test the use of vitamin D (in its different forms) as a treatment for long COVID (post-COVID syndrome). Table S3 summarizes the main features of published trials and registered trials, such as the design, patient population, interventions, outcomes, status and findings.

As of 2025, the literature does not identify any other major RCTs that exclusively study the subject of long COVID and vitamin D. Interestingly, a vitamin D trial in acute COVID-19 (200,000 IU single-dose vs. placebo) investigating the probability of long COVID symptoms after 1 year (a post hoc study) did not show any difference [112]. In general, the existing evidence based on specific trials indicates that higher doses of vitamin D or vitamin K2 can alleviate some long COVID symptoms (such as fatigue and inflammation) as well, but with varying results, and additional large-scale trials are needed [113]. Every trial showed a good safety profile for vitamin D interventions in this population. This is the contribution of long COVID to the vitamin D debate: in addition to the results regarding its effects on acute infection, adequate vitamin D could also be relevant to recovery after the virus. This is an ongoing research field, and future studies will more clearly determine whether vitamin D should specifically be used as a method to reduce the long-COVID-19 burden.

Despite its clinical relevance, the current evidence linking vitamin D status to long COVID is subject to important limitations. Most available studies are observational in design, rely on relatively small cohorts, and are susceptible to residual confounding and reverse causality [73]. In many cases, vitamin D status was assessed after acute infection or during follow-up, limiting the ability to determine whether deficiency preceded the development of long COVID or resulted from prolonged illness, reduced mobility, or systemic inflammation [114]. Furthermore, heterogeneity in long COVID definitions, symptom assessment, and follow-up duration complicates direct comparison across studies. To date, there is limited randomized controlled trial evidence demonstrating a causal role for vitamin D supplementation in preventing or treating long COVID, and existing interventional studies remain small and exploratory [73]. These limitations underscore that current findings should be interpreted cautiously and viewed as hypothesis-generating rather than definitive.

Importantly, several studies have not identified a significant association between vitamin D status and long COVID outcomes after adjustment for confounders, and interventional data remain limited to small trials with heterogeneous endpoints [73]. As such, current evidence does not allow for firm conclusions regarding a protective or therapeutic role of vitamin D in long COVID.

## 8. Clinical Guidelines and Controversies

Discussion regarding the role of vitamin D in COVID-19 and other infections has not been without criticism. Both the enthusiasm generated by observational studies and the mechanistic plausibility prompted experts to advocate mass vitamin D supplementation during the pandemic, whereas others were careful, waiting for convincing evidence from randomized trials. Clinical practice guidelines have been mostly conservative, with their recommendations stating that vitamin D is recommended for general health but not for the treatment of COVID-19. These are the existing guidelines, the meta-analytic evidence, and areas of contention.

Most health departments have focused on the importance of proper levels of vitamin D as a means to improve overall health, particularly in the winter seasons or during lockdowns, when people are not exposed to the sun. For example, the United Kingdom National Institute of Health and Care Excellence (NICE) and Public Health England recommend that adults and children over the age of 4 take vitamin D doses of 10 ug (400 IU) of daily during the fall and winter as a supplement to maintain bone and muscle health, and that at-risk populations take it year-round to prevent deficiency (e.g., those with limited sun exposure or with darker skin). This recommendation to take a daily dose of vitamin D was repeated during lockdowns in response to COVID-19 as a precautionary step to boost immune activity. However, in relation to the application of high-dose vitamin D, specifically for the prevention or treatment of COVID-19, official guidelines were conservative. In an expedited guideline that was published in late 2020, the NICE was categorically against the use of vitamin D supplements as an intervention, to either prevent or treat COVID-19, outside of clinical trial settings [48]. Likewise, the U.S. NIH COVID-19 Treatment Guidelines claim that there is no evidence either in favor of or against the use of vitamin D (or any other supplement) to prevent or treat COVID-19 [115]. Such stances are based on the rule that although vitamin D deficiency must be treated, the use of high-dose supplements cannot be recommended as a treatment for COVID-19 unless supported by facts.

Summarizing the above, meta-analyses have not consistently found a significant role of vitamin D in COVID-19 outcomes, particularly in terms of mortality [116]. This has dampened some of the initial enthusiasm. For example, a meta-analysis of RCT’s by *Frontiers* concluded that vitamin D supplementation did not significantly reduce deaths or ICU admissions [65]. Conversely, a meta-analysis in *Nutrients*, from 2024, implied a significant decrease in ICU admission and severe outcomes [88]. Clinicians and the general population can be misled by such variances in conclusions. Opponents of this claim point to the fact that most COVID-19 trials were limited by smaller sample sizes, different baseline levels of vitamin D, or different outcome measures, and therefore a meta-analysis combining them can produce conflicting outcomes based on the inclusion criteria. The other complicating factor is that some of the positive studies conducted at early stages were observational or open-label and prone to bias, and rigorous placebo-controlled trials reported less significant effects. This fact (large advantages in observational data and small or nonexistent ones in RCTs) has resulted in scientific controversy. Other researchers consider the effects of vitamin D to be real but diluted during trials through the recruitment of patients with relatively sufficient vitamin D levels or administration of the supplement too late in the progression of the disease. Some state that the observational associations were confounded or spurious, and that the largely negative results of well-controlled trials suggest that vitamin D is not a key variable in the outcomes of COVID-19 in most patients [65,92]. The reality could be somewhere in the middle: there is a high likelihood that vitamin D would be helpful in those who are deficient (and in certain situations, such as older, high-risk patients), but the widespread supplementation of all patients above a certain threshold would not result in spectacular changes.

The consensus is that vitamin D supplements are extremely safe within the recommended dosage (400–2000 IU/day) and there is very little risk of toxicity in this range. Therefore, taking adequate vitamin D is perceived by many experts as low-risk and potentially high-reward. At the beginning of the pandemic, certain clinicians established a routine of administering moderate doses (e.g., 1000–2000 IU/day or single bolus) to patients with COVID-19 to address an unidentified deficiency. Nonetheless, more controversy has surrounded the use of mega-doses (e.g., 100,000+ IU boluses) [117,118,119]. While generally safe, a few trials suggested that extremely high doses might transiently overshoot optimal levels or even paradoxically suppress aspects of immune function. The optimal dosing regimen is controversial: low doses taken continuously can provide superior immune priming, but boluses are noted to provide rapid repletion. The meta-analytic data suggest that a daily dosing method is more effective in infection prevention [39]. Based on this, some experts suggest that one should take a daily dose for maintenance instead of waiting to be treated with a high dose when infected.

During the onset of the pandemic, leading scientists published appeals urging authorities to raise daily vitamin D recommendations (e.g., to 800–2000 IU) to optimize the immune system of the population [48,120]. They stated that since vitamin D was already known to be safe, and the evidence was suggestive, lack of action may cost lives in the short term, which was captured in such headlines as “Vitamin D and COVID-19: this is no time for procrastination” [121]. On the contrary, some skeptics (including some endocrinologists and experts in evidence-based medicine) pointed to previous hype regarding vitamin D’s effect in other illnesses (cancer, cardiovascular disease) that was not borne out in trials. This dynamic produced a polarized discussion: advocates highlighting every positive study or mechanistic insight, and skeptics emphasizing null results and warning against over-supplementation.

As of 2025, there is an emerging consensus, which is the middle ground. The general recommendation of clinical guidelines is that people should maintain a sufficient level of vitamin D in the body in order to promote general health [48]. Most guidelines, in the context of COVID-19, do not go further to recommend high-dose vitamin D as a treatment, since trial findings are inconsistent. Nevertheless, they admit that it is logical to prevent vitamin D deficiency, which indirectly contributes to immune resiliency. Similarly, in the case of influenza and other respiratory infections, such organizations as the Institute of Medicine or the Endocrine Society also recommend the Daily Reference Intake (DRI) or Recommended Dietary Allowance (RDA) of vitamin D (600–800 IU in adults) and increased doses in deficient individuals, which may have certain positive effects in terms of preventing respiratory infections [122]. The use of vitamin D as a complementary immune-supportive intervention (and not in the place of vaccines or antivirals) is popularly embodied in guidelines [116]. In the meantime, studies are ongoing: mega-RCTs (e.g., CORONAVIT in the United Kingdom, the VIVID trial in the United States) are in progress, which could provide a final answer to the question of whether targeted vitamin D supplementation can prevent respiratory diseases or improve the outcomes of COVID-19. To conclude, the vitamin D controversy is not so much about safety, on which there is agreement, but rather the extent of the potential benefit. Although the impact of vitamin D on infections does appear to be small, since it is cheap and there is minimal risk of adverse effects on human health, it is perceived by many as a sensible measure for the population to take. The continuing discussion makes it even clearer that science-based guidelines should keep incorporating new evidence; the only aspect that is not in doubt is that vitamin D plays a crucial role in supporting the immune system and that vitamin D deficiency should be prevented at all costs, whether there is a pandemic or not.

## 9. Limitations and Evidence Gaps

Much of the evidence is observational in nature; hence, causality is not established. Low vitamin D levels may merely be associated with other risk factors (age, obesity, comorbidities), so confounders may explain the associations. This complicates the determination of whether vitamin D is protective itself or a marker of ill health. Acute infection and systemic inflammation have been reported to be associated with transient reductions in circulating 25(OH)D concentrations, possibly reflecting redistribution, altered binding protein levels, or negative acute-phase responses, although this phenomenon remains debated and is not universally accepted [123,124]. This makes it difficult to interpret studies that reported low vitamin D levels in severe cases. Randomized trials of vitamin D show mixed data, with some indicating effects (e.g., fewer ICU admissions) and others not. Heterogeneity is high—different trials varied in doses, timing, baseline vitamin D status and patients. Several RCTs were small and underpowered, and earlier positive studies were not rigorously blinded, which reduces the reliability of their results. Not all systematic reviews and meta-analyses obtained the same results—some show less severe outcomes with vitamin D, and others show no obvious mortality advantage. This contradiction reflects differences in the quality and methodologies of the included studies. It is possible to mistakenly infer real effects when pooling very different trials (hospitalized and outpatients, high-dose bolus and daily dosing) and draw unclear conclusions. Evidence regarding vitamin D levels and long COVID is developing but preliminary. Deficiency has been associated with increased risk of long COVID, but the cohorts were small and observational. No RCTs have evaluated whether vitamin D supplementation can definitively prevent or treat long COVID symptoms, which is also an evident gap that will have to be closed by ongoing trials.

It is not clear what dose and regimen of vitamin D is most beneficial to the immune system. Much of the research did not stratify the results based on the attained vitamin D levels; therefore, it is unclear how much one requires to protect themselves with 25(OH)D improvements. Additionally, English-language, peer-reviewed articles were reviewed in 2020–2025; it is possible that other useful data (such as non-English articles or earlier studies) were overlooked.

Clinical guidelines remain prudent because of these evidence gaps. There is a consensus that vitamin D deficiency should be avoided for good overall health, but high doses of vitamin D as a specific COVID-19 treatment are not universally supported based on the existing evidence. Thus, future studies should be conducted more decisively to go beyond correlation and to conclusively prove how vitamin D status influences COVID-19 and other respiratory diseases.

Interpretation of the available evidence requires caution. Much of the literature linking vitamin D status to COVID-19, long COVID, and influenza outcomes is observational and therefore subject to confounding, selection bias, and reverse causality. Although mechanistic studies provide biological plausibility, these findings do not establish causation at the clinical level. Accordingly, the associations described in this review should not be interpreted as evidence of direct causal effects unless supported by randomized controlled trial data.

## 10. Future Directions

Despite extensive research, several important questions regarding the role of vitamin D in respiratory viral infections remain unresolved. Future randomized controlled trials should be adequately powered and stratified by baseline serum 25(OH)D levels to clarify whether supplementation provides the greatest benefit in deficient populations. Dosing regimens, formulations (cholecalciferol versus calcifediol), timing of administration, and clinically meaningful endpoints also needed to be standardized.

In the context of COVID-19, additional trials are required to determine whether early or preventive vitamin D supplementation can reduce disease severity or long-term sequelae, including long COVID. The emerging evidence linking vitamin D deficiency to persistent post-COVID symptoms warrants targeted interventional studies assessing whether vitamin D repletion improves recovery trajectories.

For influenza and other respiratory viral infections, further research should explore optimal supplementation strategies across different age groups and risk populations, as well as interactions between vitamin D, vaccination responses, and other immune-modulating factors. Addressing these gaps will help refine evidence-based recommendations and clarify the role of vitamin D as a supportive intervention in infectious disease prevention and recovery.

## 11. Conclusions

Vitamin D plays an important role in immune regulation and host responses to respiratory viral infections. Evidence accumulated during the COVID-19 pandemic suggests that low vitamin D status is frequently associated with more severe disease outcomes, while randomized controlled trials of vitamin D supplementation have yielded heterogeneous results. Emerging observational and interventional studies further indicate that inadequate vitamin D levels may contribute to the risk and symptom burden of long COVID, although causality remains to be established. In the context of influenza, randomized trials and meta-analyses support a modest protective effect of vitamin D supplementation, particularly among individuals with low baseline vitamin D status. Overall, vitamin D should not be regarded as a standalone treatment for viral infections; however, maintaining adequate vitamin D levels represents a safe, low-cost strategy that may enhance immune resilience and complement established preventive and therapeutic measures.

## Data Availability

Data sharing is not applicable.

## Supplementary Materials

The following supporting information can be downloaded at: https://www.mdpi.com/article/10.3390/nu18040634/s1, Table S1: Summary of RCTs on Vitamin D and Influenza; Table S2: RCTs of Vitamin D or Calcifediol in COVID-19 (Treatment and Prevention); Table S3: Randomized Trials of Vitamin D for Long COVID Symptoms.

## Abbreviations

The following abbreviations are used in this manuscript:

25(OH)D	Calcifediol (25-hydroxyvitamin D)
aHR	Adjusted hazard ratio
ARI	Acute Respiratory Infection
BDG	Serum 1,3-beta-D-glucan
D_3_	Cholecalciferol
DRI	Daily Reference Intake
FEV	Forced Expiratory Volume
HCWs	Healthcare Workers
HR	Hazard Ratio
ICU	Intensive Care Unit
IL	Interleukin
ILL	Influenza-like Illness
IU	International Unit
K2	Menaquinone
LDH	Lactate dehydrogenase
LDL	low-density lipoprotein
NICE	National Institute of Health and Care Excellence
NIH	National Institute of Health
NLR	Neutrophil-to-Lymphocyte Ratio
PASC	Post-Acute Sequelae of COVID-19
PCR	Polymerase Chain Reaction
RAS	Renin–Angiotensin System
RCT	Randomized Controlled Trial
RDA	Recommended Dietary Allowance
TNF	Tumor Necrosis Factor
URI	Upper Respiratory Infection
VDR	Vitamin D Receptor
