# Supplementary material for: Vitamin D in Infectious Diseases: A Narrative Review Focusing on COVID-19, Long COVID, and Influenza
Source: Nutrients. 2026 Feb 14;18(4):634. doi: 10.3390/nu18040634 (PMC12943368; doi:10.3390/nu18040634)
Supplement: Supplementary file 1 [file nutrients-18-00634-s001.zip › nutrients-4114378-supplementary.pdf]

Table S1. Summary of RCTs on Vitamin D and Influenza

Trial (ID)	Country / Setting	Population	Vitamin D Intervention	Comparator	Primary Outcome(s)	Status	Key Findings
Urashima et al. 2010 (*UMIN000001373*) [1]	Japan; school setting	Schoolchildren (avg ~10 years old)	Vitamin D_3 1200 IU/day (oral)	Placebo (oral)	Incidence of influenza A (lab-confirmed)	Published (2010)	Influenza A occurred in 10.8% of vitamin D group vs 18.6% of placebo (RR ~0.58, *p* = 0.04). Vitamin D significantly reduced influenza A risk, especially in children not on other vit-D and older starters; also fewer asthma attacks in D group. No significant effect on influenza B.
Zhou et al. 2018 [2]	China; multicenter hospitals	Infants (≤12 months old)	High-dose vit-D_3 1200 IU/day (oral)	Low-dose vit-D_3 (likely 400 IU/day)	Incidence of influenza A (with symptom monitoring & viral load)	Published (2018)	High-dose vitamin D halved influenza A risk: 43 cases vs 78 in low-dose group (χ^2 = 14.63, *p* = 0.0001). High-dose D infants also had shorter fever, cough, wheezing duration and faster decline in viral load. No increase in adverse events; 1200 IU/day deemed effective and safe for infants.
Loeb et al. 2019 (*NCT01705314*) [3]	Vietnam (McMaster Univ. collaboration); community	Healthy children & adolescents (3–17 years)	Vitamin D_3 14,000 IU weekly (≈2000 IU/day)	Placebo (weekly)	RT-PCR–confirmed influenza (A or B); co-primary: non-influenza viral ARIs	Published (2019)	No reduction in influenza: 7.7% vs 6.6% infected in D vs placebo (HR 1.18, 95% CI 0.79–1.78, *p* > 0.4). However, vitamin D reduced non-influenza respiratory viral infections (22.5% vs 28.5%; HR 0.76, 95% CI 0.61–0.94). Overall respiratory infections (including flu) were slightly lower in D group (HR 0.81, *p* ~0.05).

DO-IT Trial (Aglipay et al. 2017) (*NCT01419262*) [4]	Canada; primary care clinics (Toronto)	Healthy toddlers & children (1–5 years)	High-dose vit-D_3 2000 IU/day	Standard-dose vit-D_3 400 IU/day	Number of laboratory-confirmed URTIs over winter (incl. influenza)	Published (2017)	No significant difference in winter URI incidence: 1.05 vs 1.03 infections per child (high vs standard dose; IRR ~0.97, 95% CI 0.80–1.16). No difference in time to first infection or parent-reported illnesses. High-dose did not reduce influenza infections compared to 400 IU/day (findings do not support routine high-dose for URI prevention).
ViDiFlu (Martineau et al. 2015) (*NCT01069874*) [5]	United Kingdom; sheltered housing clusters (London)	Older adults (residents ≥65) and their caregivers	Intermittent high-dose vit-D_3 (Resident: 2.4 mg every 2 months ≈ 96,000 IU + daily 400 IU; Caregiver: 3 mg every 2 months)	Placebo (every 2 months) + daily 400 IU for residents (caregivers' placebo only)	Time to first acute respiratory infection (ARI; upper or lower, incl. influenza)	Published (2015)	No preventive benefit: Time to first ARI was similar (aHR 1.18, p = 0.42). In fact, high-dose D was associated with higher URI risk (aHR 1.48, p = 0.039) and longer URI symptom duration (median 7 vs 5 days). No effect on lower respiratory infections. Conclusion: Adding bolus-dose D_3 to a low-dose regimen did not prevent ARIs (no specific reduction in influenza).
Arihiro et al. 2019 [6]	Japan; hospital clinics	Adults with Inflammatory Bowel Disease (Crohn's or UC)	Vitamin D_3 500 IU/day (oral)	Placebo	Incidence of seasonal influenza (winter); secondary: any upper respiratory infection (URI)	Published (2019)	No difference in influenza incidence between vitamin D and placebo groups. However, vitamin D patients had significantly fewer non-influenza URIs (RR 0.59, p = 0.042), especially if baseline 25(OH)D < 20 ng/mL. Notably, in ulcerative colitis patients, disease activity index

							worsened in the D group (an unexpected finding).
Huang et al. 2022 [7]	Taiwan; daycare centers	Preschool children (2–5 years)	Vitamin D_3 2000 IU/day for 1 month	Placebo (for 1 month)	Incidence of influenza (and enterovirus) in 6-month follow-up	Published (2022)	Trend toward fewer influenza cases with vitamin D (84% relative risk reduction vs placebo), but this did not reach statistical significance (infection probability lower in D group, p = 0.055). No effect on enterovirus incidence. All children on vitamin D achieved 25(OH)D >30 ng/mL. Authors conclude vitamin D *may* have a small preventive effect against influenza in preschoolers.
van Helmond et al. 2023 (*NCT04596657*) [8]	United States of America; tertiary hospital (New Jersey)	Healthcare workers (hospital staff)	Vitamin D_3 5000 IU/day for 9 months	No supplementation (observational control)	Influenza-like illness (ILI) incidence (self-reported, with COVID testing)	Published (2023)	Lower ILI rate with vitamin D: 5000 IU daily reduced overall ILI incidence in HCWs (incidence rate difference –1.7×10^–4 per person-day vs control, p = 0.015). Specifically, non-COVID ILIs were significantly reduced (p = 0.038), while COVID-19 infection rates were not significantly different. Suggests daily vitamin D_3 can help prevent influenza-like illness in frontline workers.
"Clinica NOVA" Trial 2021 (*NCT04810949*) [9]	Latin America (e.g. Hospital Clínica NOVA)	Health personnel (adults, ≥18) with baseline 25(OH)D >20 ng/mL	Vitamin D_3 supplementation (dose not public; presumably daily high-dose)	No supplementation + standard care	Respiratory infections (COVID-19 and influenza) incidence	Completed (2022; results pending)	A Phase 3 trial launched in 2021 to test if vitamin D prevents COVID-19 and influenza in healthcare staff. Enrollment ~877; now completed (status: active, not recruiting). Awaiting results –

							preliminary reports (preprint) suggest vitamin D may lower COVID-19 infection rates, but no peer-reviewed data yet on influenza outcomes.

Abbreviations: vitamin D3 = cholecalciferol; 25(OH)D = calcifediol (25-hydroxyvitamin D); RCT = randomized controlled trial; PCR = Polymerase chain Reaction; ARI = acute respiratory infection; URI = upper respiratory infection ; aHR = adjusted Hazard Ratio; ILL = Influenza-like illness; ~~P – published~~; ~~C – completed~~. **Footnote:** High dose ≥4,000 IU/day or equivalent; bolus ≥50,000 IU intermittently. Serum 25(OH)D reported in ng/mL

Table S2. RCTs of Vitamin D or Calcifediol in COVID-19 (Treatment and Prevention)

Trial (Identifier)	Country	Population	Intervention (Vitamin D form & dose)	Comparator	Primary Outcome(s)	Status	Key Findings
Cordoba Calcifediol Pilot (no reg. ID) [10]	Spain	Hospitalized COVID-19 patients (N=76)	*Calcifediol* (oral 25(OH)D₃): 0.532 mg on admission, then 0.266 mg on day 3, 7, and weekly	No calcifediol (standard care)	ICU admission, mortality	Published (2020)	ICU admissions: 2% vs 50% in control (1 of 50 vs 13 of 26; p<0.001). No deaths in calcifediol group vs 2 in control. Conclusion: high-dose calcifediol significantly reduced ICU need.
Murai et al. (NCT04449718) [11]	Brazil	Hospitalized patients, moderate-severe COVID (N=240)	*Vitamin D₃* (cholecalciferol) : single oral 200,000 IU dose	Placebo	Hospital length of stay	Published (2021)	No significant difference in length of stay (median 7 days both groups). No significant differences in mortality, ICU admission, or ventilation need. High-dose D3 did not improve outcomes.
SHADE (NCT04459247) [12]	India	Mild/asymptomatic COVID-19, vitamin D–deficient (N=40)	*Vitamin D₃*: 60,000 IU daily for 7 days (oral nano-liquid) (target	Placebo	SARS-CoV-2 PCR negativity by day 21	Published (2022)	62.5% vs 20.8% of patients became PCR-negative by day 21 in vitamin D vs placebo (p=0.018). Vitamin

			25(OH)D >50 ng /mL)				D group had greater drop in fibrinogen; supports faster viral clearance with high-dose D.
COVIT-TRIAL (NCT04344041) [13]	France	Older (≥65) at-risk adults with COVID-19 (hospital/nursing home, N=254)	*Vitamin D₃*: single 400,000 IU oral dose within 72 hrs of diagnosis	50,000 IU single dose (active control)	14-day mortality	Published (2022)	14-day mortality 6% high-dose vs 11% standard-dose (HR 0.39, *p*=0.049). 28-day mortality showed no significant difference (15% vs 17%, *p*=0.29). High-dose D3 improved short-term survival with no safety issues.
Sabico et al. (no reg. ID) [14]	Saudi Arabia	Mild-to-moderate COVID-19 patients (hospitalized isolation, N=69)	*Vitamin D₃*: 5,000 IU/day oral for 2 weeks (vs. 1,000 IU/day in control)	1,000 IU/day vitamin D₃	Time to symptom recovery	Published (2021)	5,000 IU group achieved higher 25(OH)D and had faster resolution of cough (median ~6.2 vs 9.1 days, *p*≈0.04) and loss of taste (11.4 vs 16.9 days, *p*≈0.035) than 1,000 IU group. No significant differences in inflammatory markers between groups.
Maghbooli et al. (no reg. ID) [15]	Iran	Hospitalized COVID-19 patients, vitamin D–deficient (<30 ng/mL, N=106)	*Calcifediol* (oral 25(OH)D₃): extended-release formula (doses titrated to raise 25(OH)D ≥50 ng/mL)	Placebo	Correction of 25(OH)D levels; blood neutrophil-to-lymphocyte ratio (NLR)	Published (2021)	25(OH)D levels ≥30 ng/mL achieved in 100% of treated vs 12.5% of placebo by 60 days. Calcifediol group had increased lymphocytes and lower NLR (immune marker). Trends toward lower ICU duration,

							ventilation, and mortality in calcifediol group, but differences not statistically significant.
REsCue (NCT04551911) [16]	United States of America	Symptomatic COVID-19 outpatients (mild-moderate, N=160 analyzed)	*Calcifediol* (extended-release capsule): 300 µg daily on days 1–3, then 60 µg daily on days 4–27.	Placebo	Time to resolution of 5 key symptoms	Published (2023)	Achieved 25(OH)D ≥50 ng/mL in 81% of treated vs 15% placebo. Primary outcome: no significant difference in time to symptom resolution overall (HR≈0.98, *p*=0.92). Post-hoc per-protocol analysis suggested ~4 days faster resolution of respiratory symptoms in those achieving high 25(OH)D [64]. No safety issues (no hypercalcemia).
CORONAVIT (NCT04579640) [17]	United Kingdom	General population (age ≥16, not on vit D, N=6,200)	*Vitamin D₃* "test-and-treat": offer 25(OH)D home test; if <75 nmol/L, supplement for 6 months (either 800 IU/day or 3,200 IU/day)	No testing or supplementation	Incidence of acute respiratory infection (ARI); COVID-19 incidence	Published (2022)	No significant reduction in ARI: 5.7% (800 IU) and 5.0% (3200 IU) vs 4.6% in no-vitamin D group (OR not significant). COVID-19 incidence also not significantly different (3.6% and 3.0% vs 2.6% in controls). Conclusion: population-level vit D test-and-supplement strategy did *not* reduce risk of respiratory

							infections or COVID.
CLOC (Cod Liver Oil trial) (NCT04609423) [18]	Norway	General adults (age 18–75, winter months, N=34,601)	*Cod liver oil* (low-dose vit D source): 5 mL daily (≈400 IU vit D₃) for up to 6 months.	Placebo (5 mL corn oil)	SARS-CoV-2 infection rate; "serious" COVID-19; other ARIs	Published (2022)	No differences between cod liver oil vs placebo in any primary endpoint. PCR-confirmed COVID occurred at similar rates; "serious COVID-19" cases were 121 vs 101 (cod liver vs placebo); incidence of other acute respiratory infections was also similar (e.g. ~3964 vs 3834 with ≥1 ARI) [66]. Conclusion: 400 IU/day vitamin D (via cod liver oil) did not prevent COVID-19 or other ARIs.
PROTECT (NCT04483635) [19]	Canada	Healthcare workers (high-exposure settings, N≈*planned 1,980*)	*Vitamin D₃*: one-time 100,000 IU oral loading + 10,000 IU weekly maintenance.	Placebo loading + weekly placebo	Incidence of lab-confirmed COVID-19 infection (HCWs).	Finished (2023)	Trial stopped early due to poor recruitment. (Planned to assess if high-dose D could reduce infection or severity in HCWs. No final efficacy data reported due to early termination).
VIVID (NCT04536298) [20]	United States of America/ Mongolia	Recently diagnosed COVID-19 cases and their household contacts (cluster design, *target* N=2,700).	*Vitamin D₃*: COVID+ cases get high-dose D (initial bolus + 3,200 IU/day for 4 weeks); their household contacts likewise supplemented.	Placebo (for both patients and contacts)	COVID+ cases: composite of hospitalization or death; Household contacts: SARS-CoV-2	Ongoing (planned in 2020–2024)	Not yet published (pragmatic cluster-RCT underway). Aim is to test if loading + daily 3,200 IU D₃ early in COVID can reduce progression (severe outcomes)

			infection rate.	and if prophylactic D can prevent transmission in households. Results pending as of 2025.

Abbreviations: vitamin D3 = cholecalciferol; 25(OH)D = calcifediol (25-hydroxyvitamin D); RCT = randomized controlled trial; ICU = intensive care unit; PCR = Polymerase chain Reaction; ARI = acute respiratory infection; HCWs = Healthcare workers; NLR = Neutrophil-to-Lymphocyte Ratio; ~~P – published, F – finished; O – ongoing.~~
Footnote: High dose ≥4,000 IU/day or equivalent; bolus ≥50,000 IU intermittently. Serum 25(OH)D reported in ng/mL

Table S3. Randomized Trials of Vitamin D for Long COVID Symptoms

Trial (Location)	Population	Intervention vs. Control	Primary Outcomes	Status / Findings
Atieh et al., 2025 *Vitamins K2 and D3 for Long COVID* (United States of America – Cleveland, OH) [21]	151 adults (median age 46; ~71% female) with ≥2 moderate long COVID symptoms ≥3 months post-infection.	Daily Vitamin K2 (MK-7) 240 µg + Vitamin D_3 2000 IU (co-formulated) for 24 weeks vs. Standard of Care (no placebo).	Changes in long COVID symptom burden (RECOVER LC symptom index ≥12, number of symptoms) and inflammatory/gut biomarkers at 24 weeks.	Published (Completed) – Vitamin K2+D_3 group showed significant improvement: reduction in proportion of patients with high symptom index (7.1% decrease *vs* 7.2% increase in control, $p = 0.01$) and stable symptom count (vs increase in control, $p = 0.03$) [83]. Also saw decreases in oxidized LDL and inflammatory markers (sTNF-RI, sCD163) and fungal translocation marker BDG with K2+D_3. Authors conclude combined K2+D_3 safely improved long COVID symptoms and inflammation compared to standard care.
Charoenporn et al., 2024 *High-Dose Vitamin D in Post-COVID*	80 adults with post-COVID fatigue or neuropsychiatric	Vitamin D_3 60,000 IU weekly (oral cholecalciferol,	Symptom scales: fatigue (Chalder Fatigue Scale, CFQ-11), mental health	Published (Completed) – The vitamin D group showed significant

(Thailand – Thammasat Univ.) [22]	symptoms (persistent symptoms after recovery; all had fatigue and/or cognitive, mood issues). Randomized 1:1.	high-dose) for 8 weeks vs. Placebo weekly for 8 weeks.	(Depression Anxiety Stress Scale, DASS-21), sleep quality (PSQI), cognitive function tests (ACE-III, Trail Making A/B). Also, inflammatory markers (IL-6, CRP).	improvements in fatigue scores (CFQ-11, –3.5 vs placebo, $p = 0.024$), anxiety scores (DASS-Anxiety –2.0, $p = 0.011$), and cognitive function (ACE +2.1 points, $p = 0.012$). No significant differences in depression scores, sleep quality, or IL-6/CRP levels [84]. No serious adverse events occurred. Conclusion: High-dose vitamin D improved fatigue, anxiety, and cognitive symptoms in long COVID with minimal side effects.
Caballero-García et al., 2021 *Vitamin D for Muscle Recovery* (Spain – Soria) [23]	30 elderly male patients (post-acute COVID-19 recovery phase; ~mean 70 y, all men) in a primary care setting, ≥3 months after infection (persistent weakness).	Vitamin D_3 2,000 IU/day (oral cholecalciferol) for 6 weeks vs. Placebo daily for 6 weeks (double-blind).	Muscle recovery indicators (serum muscle enzymes: creatine kinase, LDH, etc.), respiratory function (FEV_1, peak flow), and physical performance (6-minute walk test).	Published (Completed) – Vitamin D group had lower muscle damage markers: serum creatine kinase normalized (decreased to optimal levels) with vitamin D vs. remaining elevated in placebo. Patients on vitamin D reported feeling better, though no statistically significant improvement was seen in 6-minute walk distance or lung function vs. placebo. Authors suggest vitamin D may aid muscle recovery after COVID (reducing muscle catabolism), potentially improving

				quality of life in long COVID convalescents.
Magnesium + Vitamin D for Post-COVID (NCT05630339) (Mexico – IMSS Health Services) [24] Study Details | NCT05630339 | Magnesium and Vitamin D Combination for Post-COVID Syndrome | ClinicalTrials.gov	150 adults with post-COVID syndrome (long COVID symptoms, any gender, ≥18 y) enrolled after acute COVID recovery (exact inclusion criteria per registry)	Magnesium chloride 1.3 g/day total (650 mg capsule × 2, providing ~300 mg elemental Mg) + Vitamin D_3 4,000 IU/day, orally for 4 months vs. Placebo (inert) daily for 4 months (Double-blind, placebo-controlled).	Improvement in long COVID symptoms over 4 months (fatigue, post-COVID wellness); baseline and 4-month lab measures of 25(OH)D, magnesium levels, metabolic panel. (Likely primary outcome: symptom score or proportion improved).	Completed (Results pending) – *Status:* Trial completed in 2023. As of now no results are posted and no peer-reviewed publication yet. Awaiting findings on whether Mg + D supplementation improved fatigue, anxiety, depression and overall post-COVID symptomatology compared to placebo.

Abbreviations: vitamin D3 = cholecalciferol; 25(OH)D = calcifediol (25-hydroxyvitamin D); vitamin K2 = menaquinone; RCT = randomized controlled trial; CRP = C reactive protein; IL = interleukin; LDL - low-density lipoprotein; BDG = Serum 1,3-beta-D-glucan; LDH = Lactate dehydrogenase; FEV = Forced Expiratory Volume; Footnote: High dose ≥4,000 IU/day or equivalent; bolus ≥50,000 IU intermittently. Serum 25(OH)D reported in ng/mL.

Supplementary file Reference

1. Urashima, M.; Segawa, T.; Okazaki, M.; Kurihara, M.; Wada, Y.; Ida, H. Randomized trial of vitamin D supplementation to prevent seasonal influenza A in schoolchildren. *Am. J. Clin. Nutr.* **2010**, *91*, 1255–1260.
2. Zhou, J.; Du, J.; Huang, L.; Wang, Y.; Shi, Y.; Lin, H. Preventive Effects of Vitamin D on Seasonal Influenza A in Infants: A Multicenter, Randomized, Open, Controlled Clinical Trial. Pediatr. Infect. Dis. J. 2018, 37, 749–754.
3. Loeb, M.; Dang, A.D.; Thiem, V.D.; Thanabalan, V.; Wang, B.; Nguyen, N.; Mai Tran, H.T.; Luong, T.; Singh, P. et al. Effect of Vitamin D supplementation to reduce respiratory infections in children and adolescents in Vietnam: A randomized controlled trial. Influenza Other Respir. Viruses 2019, 13, 176–183.
4. Aglipay, M.; Birken, C.S.; Parkin, P.C.; Loeb, M.; Thorpe, K.; Chen, Y.; Lapuacis, A.; Mamdani, M.; Macarthur, C.; Hoch, J. et al. Effect of High-Dose vs Standard-Dose Wintertime Vitamin D Supplementation on Viral Upper Respiratory Tract Infections in Young Healthy Children. *JAMA* **2017**, *318*, 245–254.
5. Martineau, A.R.; Hanifa, Y.; Witt, K.D.; Barnes, N.; Hooper, R.; Patel, M.; Stevens, N.; Enayat, Z.; Balayah, Z.; Syed, A. et al. Double-blind randomised controlled trial of vitamin D3 supplementation for the prevention of acute respiratory infection in older adults and their carers (ViDiFlu). Thorax 2015, 70, 953–960.
6. Arihiro, S.; Nakashima, A.; Matsuoka, M.; Suto, S.; Uchyiama, K.; Kato, T.; Mitobe, J.; Komoike, N.; Itagaki, M.; Miyakawa, Y. et al. Randomized Trial of Vitamin D Supplementation to Prevent Seasonal Influenza and Upper Respiratory Infection in Patients With Inflammatory Bowel Disease. Inflamm. Bowel Dis. 2019, 25, 1088–1095.

7. Huang, Y.N.; Chi, H.; Chiu, N.C.; Huang, C.Y.; Li, S.T.; Wang, J.Y.; Huang, D.T. A randomized trial of vitamin D sup-plementation to prevent seasonal influenza and enterovirus infection in children. J. Microbiol. Immunol. Infect. 2022, 55, 803–811.
8. van Helmond, N.; Brobyn, T.L.; LaRiccia, P.J.; et al. Vitamin D3 Supplementation at 5000 IU Daily for the Prevention of Influenza-like Illness in Healthcare Workers: A Pragmatic Randomized Clinical Trial. Nutrients Microorganisms 2023, 15, 180.
9. Wang, X.; Li, J.; Liu, H.; Hu, X.; Lin, Z.; Xiong, N. SARS-CoV-2 versus Influenza A Virus: Characteristics and Co-Treatments. Microorganisms 2023, 11, 580.
10. Entrenas-Castillo, M.; Entrenas-Costa, L.M.; Vaquero-Barrios, J.M.; et al. Effect of calcifediol treatment and best available therapy versus best available therapy on intensive care unit admission and mortality among patients hospitalized for COVID-19: A pilot randomized clinical study. J. Steroid Biochem. Mol. Biol. 2020, 203, 105751.
11. Murai, I.H.; Fernandes, A.L.; Sales, L.P.; et al. Effect of a Single High Dose of Vitamin D3 on Hospital Length of Stay in Patients With Moderate to Severe COVID-19: A Randomized Clinical Trial. JAMA. 2021, 325, 1053–1060.
12. Rastogi A, Bhansali A, Khare N, Suri V, Yaddanapudi N, Sachdeva N, Puri GD, Malhotra P. Short term, high-dose vitamin D supplementation for COVID-19 disease: a randomised, placebo-controlled, study (SHADE study). Postgrad Med J. 2022, 98, 87-90.
13. Annweiler, C.; Beaudenon, M.; Gautier, J.; et al. COVIT-TRIAL study group. High-dose versus standard-dose vitamin D supplementation in older adults with COVID-19 (COVIT-TRIAL): A multicenter, open-label, randomized controlled su-periority trial. PLoS Med Endocr Pract.. 2022, 19, e1003999.
14. Sabico, S.; Enani, M.A.; Sheshah, E.; Aljohani, N.J.; Aldisi, D.A.; Alotaibi, N.H.; Alshingetti, N.; Alomar, S.Y.; Alnaami, A.M.; Amer, O.E.; et al. Effects of a 2-Week 5000 IU versus 1000 IU Vitamin D3 Supplementation on Recovery of Symp-toms in Patients with Mild to Moderate Covid-19: A Randomized Clinical Trial. Nutrients 2021, 13, 2170. https://doi.org/10.3390/nu13072170.
15. Maghbooli, Z; Sahraian, MA; Jamalimoghadamsiahkali, S; et al. Treatment With 25-Hydroxyvitamin D3 (Calcifediol) Is Associated With a Reduction in the Blood Neutrophil-to-Lymphocyte Ratio Marker of Disease Severity in Hospitalized Patients With COVID-19: A Pilot Multicenter, Randomized, Placebo-Controlled, Double-Blinded Clinical Trial. Endocr Pract. 2021, 27, 1242-1251.
16. Bishop, CW; Ashfaq, A; Melnick, JZ; et al. REsCue trial: Randomized controlled clinical trial with extended-release calci-fediol in symptomatic COVID-19 outpatients. Nutrition 2023, 107, 111899.
17. Jolliffe, D.A.; Holt, H.; Greenig, M.; et al. Effect of a test-and-treat approach to vitamin D supplementation on risk of all cause acute respiratory tract infection and covid-19: phase 3 randomised controlled trial (CORONAVIT). BMJ 2022, 378, e071230.
18. Brunvoll SH, Nygaard AB, Ellingjord-Dale M, Holland P, Istre MS, Kalleberg KT, Søraas CL, Holven KB, Ulven SM, Hjartåker A, Haider T, Lund-Johansen F, Dahl JA, Meyer HE, Søraas A. Prevention of covid-19 and other acute respiratory infections with cod liver oil supplementation, a low dose vitamin D supplement: quadruple blinded, randomised placebo controlled trial. BMJ. 2022 Sep 7;378:e071245.
19. Ducharme, FM; Tremblay, C; Golchi, S; et al. Prevention of COVID-19 with oral vitamin D supplemental therapy in es-sential healthcare teams (PROTECT): protocol for a multicentre, triple-blind, randomised, placebo-controlled trial. BMJ Open. 2023, 13, e064058.
20. Wang, R; DeGruttola, V; Lei, Q; et al. The vitamin D for COVID-19 (VIVID) trial: A pragmatic cluster-randomized design. Contemp Clin Trials. 2021, 100, 106176.
21. Atieh O, Daher J, Durieux JC, Abboud M, Labbato D, Baissary J, Koberssy Z, Ailstock K, Cummings M, Funderburg NT, McComsey GA. Vitamins K2 and D3 Improve Long COVID, Fungal Translocation, and Inflammation: Randomized Con-trolled Trial. Nutrients. 2025 Jan 16;17(2):304.
22. Charoenporn, V.; Tungsukruthai, P.; Teacharushatakit, P.; et al. Effects of an 8-week high-dose vitamin D supplementation on fatigue and neuropsychiatric manifestations in post-COVID syndrome: A randomized controlled trial. Psychiatry Clin. Neurosci. 2024, 78, 595–604.

23. Caballero-García, A.; Pérez-Valdecantos, D.; Guallar, P.; et al. Effect of vitamin D supplementation on muscle status in old patients recovering from COVID-19 infection. Medicina (Kaunas) 2021, 57, 1079.
24. Study Details | NCT05630339 | Magnesium and Vitamin D Combination for Post-COVID Syndrome | ClinicalTrials.gov. https://clinicaltrials.gov/study/NCT05630339 (Accessed on 21st of December, 2025).
